# Equilibrium in soil respiration across a climosequence indicates its resilience to climate change in a glaciated valley, western Himalaya

**DOI:** 10.1038/s41598-021-02199-x

**Published:** 2021-11-29

**Authors:** Pankaj Tiwari, Pamela Bhattacharya, Gopal Singh Rawat, Gautam Talukdar

**Affiliations:** Wildlife Institute of India, Dehradun, Uttarakhand 248001 India

**Keywords:** Climate-change impacts, Environmental impact

## Abstract

Soil respiration (SR), a natural phenomenon, emits ten times more CO_2_ from land than anthropogenic sources. It is predicted that climate warming would increase SR in most ecosystems and give rise to positive feedback. However, there are uncertainties associated with this prediction primarily due to variability in the relationship of SR with its two significant drivers, soil temperature and moisture. Accounting for the variabilities, we use a climosequence in Himalaya with a temperature gradient of ~ 2.1 °C to understand the variations in the response of SR and its temperature sensitivity to climate change. Results indicate an equilibrium in SR ranging from 1.92 to 2.42 µmol m^−2^ s^−1^ across an elevation gradient (3300–3900 m) despite its increased sensitivity to temperature (Q_10_) from 0.47 to 4.97. Additionally, moisture reduction towards lower elevation weakens the temperature-SR relationship. Finally, soil organic carbon shows similarities at all the elevations, indicating a net-zero CO_2_ flux across the climosequence. The findings suggest that as the climate warms in this region, the temperature sensitivity of SR reduces drastically due to moisture reduction, limiting any change in SR and soil organic carbon to rising temperature. We introduce an equilibrium mechanism in this study which indicates the resilient nature of SR to climate change and will aid in enhancing the accuracy of climate change impact projections.

## Introduction

Soil respiration (SR) is the process of CO_2_ emission from soil that originates mainly from microbial and root respiration. This emission is approximately tenfold higher than fossil fuel combustion^1^ and accounts for about ~ 90 PtG C emissions per year^2,3^. Variations in atmospheric CO_2_ are linked with climate-induced changes in SR, and even a tiny shift in the latter may have a profound impact on the global carbon balance^4^. Climate warming is likely to increase SR and give rise to positive feedback as studies report an exponential relationship between SR and temperature^5–7^. However, the strength of the temperature-SR relationship is variable across different habitats and is often decided by moisture availability^8^. High soil moisture impedes soil organic carbon decomposition and CO_2_ production by limiting aeration and thus O_2_ diffusivity into the soil^9^. In contrast, low water availability stresses microbial activities and translocation of nutrients to root, constraining respiration^10^. Moisture may also regulate the sensitivity of SR to temperature changes^7^.

An exponential temperature-SR relationship or Q_10_ generally indicates the temperature sensitivity of SR. Q_10_ refers to the response magnitude of SR to a 10 °C increase in temperature and is an index to forecast the feedback intensity of SR to the rising temperature^11^. Q_10_ of SR has been used as a constant of 2 in several biogeochemical models despite its high variability across different ecosystems. For instance, Chen et al.^12^ reported a wide range of Q_10_ from 1.1 to 13.5 across various biomes. In addition, studies from Nyaiqentanglha Mountains and the eastern Daba mountains observe an increasing Q_10_ with an increase in elevation^7,13^.

Elevation gradients in the mountains show substantial shifts in climatic (climosequence) and biotic characteristics over short geographic distances. Thus, these gradients are considered "natural experiments" to understand the environmental change impacts on ecosystem functioning over a considerable time scale and may be used to proxy for climate change^14^. However, limited studies have taken advantage of these gradients to understand the fate of SR response to climate change. Therefore, we aimed to (i) assess the response of SR and its temperature sensitivity across a climosequence; (ii) examine the dynamics in temperature-SR and moisture-SR relationships across the climate gradient; and (iii) quantify soil organic carbon in these gradients to assess shifts in the carbon pool under climate change.

We selected six elevations from 3300 to 3900 m encompassing subalpine forest, alpine scrub, and alpine meadow with a temperature gradient of ~ 2.1 °C. The sites were situated in a protected area in the western part of Himalaya where livestock grazing is banned, and human interference is minimal (Supplementary Fig. S1 & Supplementary Table S1 online). We periodically measured SR, temperature, moisture, and organic carbon during the growing season of 2019. We then constructed exponential and linear regression models to assess the relationship strength of SR and Q_10_ of SR with temperature and moisture at each of the elevations. Finally, we propose a theoretical framework to account for the observed trends in our results. This is the first elevational study on SR from the higher elevations of Himalaya, which will refine our understanding of how the SR will respond to changing climate.

## Results and discussion

### Seasonal patterns in SR at different elevations

**Figure 1:**
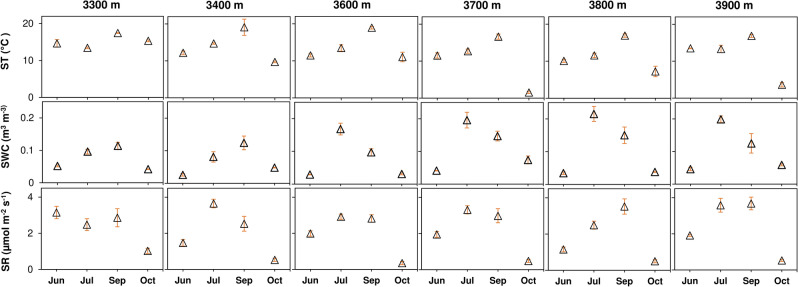
Soil temperature, moisture and, respiration at different elevations. Each triangle represents the mean environmental parameter (ST: soil temperature at 5 cm depth, SWC: volumetric soil water content at 5 cm depth, and SR: soil respiration; n = 12) ± standard error of mean during four months in 2019.

SR followed a non-linear hump-shaped curve across the sampling period, showing peaks during July–September and a decrease in October at all the elevations, overlapping with variations in soil temperature and moisture (Fig. 1). High SR during peak growing season was probably a cumulative effect of increased microbial respiration, due to temperature-induced enhanced enzymatic activity under high labile substrate availability (from plant-photosynthetic C allocates) and adequate moisture (from rainfall), and increased root respiration^15–17^. Our earlier study from the alpine meadow of this region (~ 4000 m a.s.l.) showed similar seasonal patterns in SR^18^ and is also in accord with studies from the central and north-eastern Tibetan Plateau^5,7^.

### Relationships of SR with temperature and moisture

**Figure 2:**
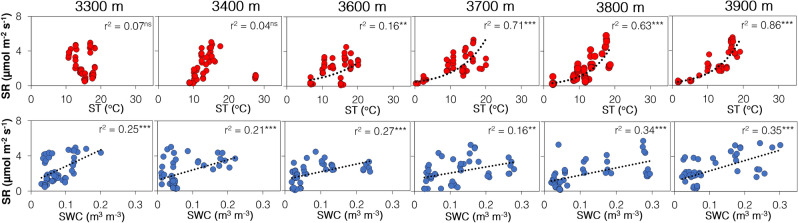
Effect of soil temperature and moisture on SR. Exponential and linear models between SR and soil temperature (ST) and SR and volumetric soil water content (SWC) at different elevations. r^2^ depicts the strength of the model significant at *** p < 0.001 and ** p < 0.01 while ns represents non-significant relationship with total data points = 48. See also Supplementary Table S2 online for intercepts, slope, and exact p values in the model.

We observed that soil temperature had no significant relation with SR in the lower elevations (3300–3400 m), but their relationships strengthened in mid (3600–3700) and higher elevations (3800–3900 m) (Fig. 2 & Supplementary Table S2 online). It is worth noting that soil moisture showed an increasing trend across elevation (Table 1) and was significantly related to SR at all the elevations (Fig. 2). Low moisture-induced drier conditions at lower elevations caused temperature insensitivity in microbes and eventually in SR^19^. In comparison, moisture at the higher elevations was adequate for the activities of both microbes and roots and, therefore, positively affected the temperature-SR relationship.

**Table 1:** Comparison of the environmental parameter at different elevations.

Elevation (m)	SOC (g Kg^-1^)	ST (°C)	SWC (m^3^ m^-3^)	SR (µmol m^-2^ s^-1^)	Q_10_ of SR
3300	33.29 ± 2.35^b^	15.21 ± 0.34^c^	0.076 ± 0.006^a^	2.38 ± 0.21^a^	0.47
3400	19.03 ± 1.91^a^	13.93 ± 0.75^bc^	0.070 ± 0.008^a^	2.05 ± 0.21^a^	1.42
3600	18.04 ± 2.68^a^	13.74 ± 0.61^bc^	0.081 ± 0.010^ab^	2.02 ± 0.17^a^	2.69
3700	26.10 ± 3.42^ab^	10.60 ± 0.86^a^	0.115 ± 0.012^b^	2.18 ± 0.20^a^	3.57
3800	19.12 ± 2.55^a^	11.49 ± 0.66^ab^	0.110 ± 0.014^ab^	1.92 ± 0.21^a^	4.97
3900	15.70 ± 0.92^a^	11.83 ± 0.78^ab^	0.106 ± 0.011^ab^	2.42 ± 0.23^a^	4.30

Growing season mean soil temperature (ST), volumetric soil water content (SWC), SR and Q_10_ of SR at different elevations. Values are mean ± standard error of mean with n = 48. Different letters indicate a significant difference in each parameter between elevations (p < 0.05).

### Temperature sensitivity (Q_10_) of SR across elevation

**Figure 3:**
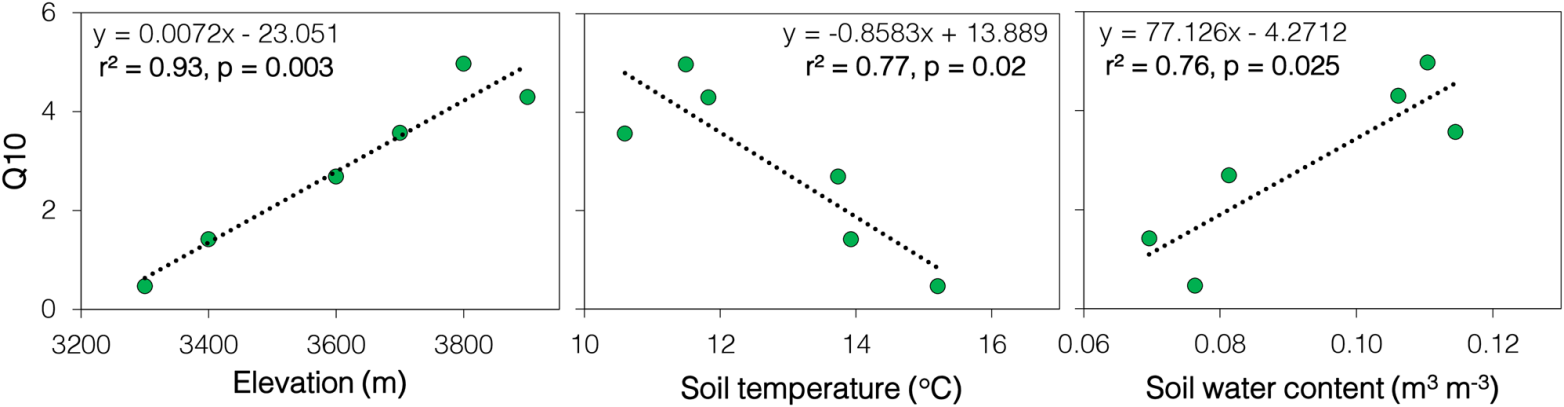
Effect of elevation, soil temperature, and moisture on the temperature sensitivity of SR. Linear models to show the significant and robust relationship (r^2^ < 0.76, p < 0.05) between Q_10_ (temperature sensitivity) of SR and elevation, soil temperature, and volumetric soil water content.

The strong and significant temperature-SR relationship at the higher elevations suggested an increase in SR under climate warming. This was also indicated by the high Q_10_ of SR at the higher elevations (Fig. 3). Contrarily,

Q_10_ decreased towards lower elevation being highest in alpine meadow at 3800–3900 m (4.64 ± 0.34) followed by alpine scrub at 3600–3700 m (3.13 ± 0.44) and subalpine forest at 3300–3400 m (0.95 ± 0.48). Soil temperature and moisture are considered the main controllers of Q_10_^12^. In our study, Q_10_ values correlated negatively with soil temperature but positively with soil moisture (Fig. 3), similar to the previous studies^7,20^. Analyzing both the observations, we can say that a consecutive rise in 2.1 °C temperature across the elevation ranges decreased the temperature sensitivity by 33% and 47%, respectively. The relationship suggests that relatively low temperature and high moisture at higher elevations made them more sensitive to climate warming than their lower counterparts. A decrease in temperature sensitivity with rising temperature has been reported in both alpine^7,21^ and sub-tropical regions^13^ and is believed to be mainly caused by a reduction in sensitivity of microbial-derived-heterotrophic respiration^21^ and partially due to reduced growth of fine roots and their respiration at lower elevations (not measured in this study)^7,13,22^. In contrast, evidences of rising heterotrophic respiration over the recent decades have also been reported^23^.

### SR, organic carbon, and the equilibrium mechanism

**Figure 4:**
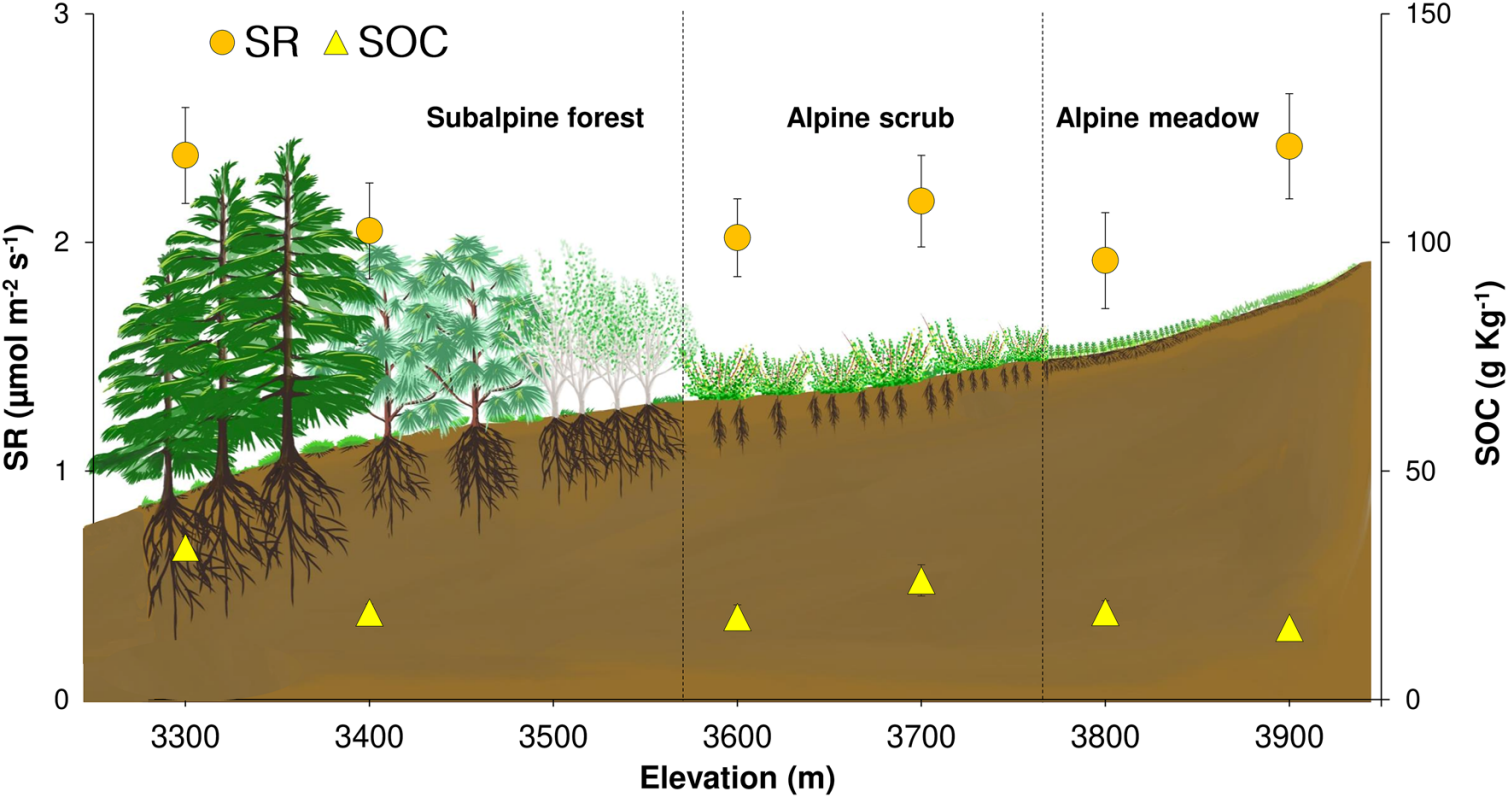
Equilibrium in SR and organic carbon across climosequence. Diagrammatic representation of a theoretical framework showing similar mean SR and soil organic carbon (SOC) at different elevations across climosequence and vegetation succession with n = 48. Note that vegetation changes from the subalpine forest (3300–3600 m) to alpine scrub (3600–3800 m) and alpine meadow (3800–4000 m) (Supplementary Table S1 online). Microsoft Excel 2019 (https://www.microsoft.com/en-us/microsoft-365/microsoft-office) and Adobe Photoshop 7.0 (https://www.adobe.com/in/products/photoshop.html) were used to prepare the graph and drawing, respectively.

Since the higher elevations were more sensitive, we expected SR to increase with an increased temperature towards lower elevations. Surprisingly, we found no significant differences in SR at any elevation (Table 1). Also, the soil organic carbon was similar across the elevation gradient (Table 1). To account for the neutrality in both SR and organic carbon with increasing temperature (decreasing elevation), we present a theoretical framework using vegetation succession across climosequence as a premise (Fig. 4). On a geological time-scale, alpine meadow comprising herbaceous and graminoid species (Supplementary Table S1 online) originated first among the three habitats^24^.

Warming-induced snow-melt and soil exposure led to forming a microclimate suitable for microbial and plant growth, eventually initiating respiration. At this point, soil moisture was adequate for their metabolic activities; however, temperature limited exponential growth^25,26^. Also, nutrient availability, both in quality and quantity, allowed only specialized microbial and plant communities^27^. Initial warming increased SR in alpine meadow and may or may not have caused soil carbon loss depending on carbon input from photosynthesis^18,28^. However, a high temperature-induced decrease in moisture and substrate availability limited SR and, in turn, carbon loss^18^. As SR acclimatized, the soil started to regain its carbon with high photosynthetic activity from increased plant growth^29^. The increasing trend in soil organic carbon is evident from 3900 to 3700 m (Table 1).

Over centuries, continued warming and soil mineralization allowed colonization by shrubs like *Artemisia* spp., *Juniperus* spp., *Rosa sericea*, *Lonicera* spp. (Supplementary Table S1 online) and transformation of alpine meadow to scrub (3500–3800 m)^30,31^. High temperature-induced low moisture in the scrub further limited the increase in SR, resulting in similar SR rates than that in the meadow^32^. Photosynthetic activity of shrubs was probably lower than that of herbs which reduced carbon input into the soil as seen from 3700 to 3400 m however was non-significant. As a result, organic carbon was similar in both alpine meadow and scrub.

Furthermore, soil mineralization and warmer climate facilitated forest succession with trees such as *Cedrus deodara, Pinus wallichiana, Populus ciliate, and Betula utilis* in the subalpine zone (3300–3500 m)^33,34^. At this stage, the temperature was sufficient for major microbial and plant communities to carry their metabolic activities. However, soil moisture continued to decrease in the forest due to high temperature and low precipitation conditions (Table 1). The dense tree canopy restricted sunlight from reaching the ground and affected the growth of ground vegetation and, in turn, below-ground fine roots (in the upper soil layer)^35^. Thus, low moisture and reduced root biomass continued to limit SR. At the same time, high litter input from forest vegetation balanced any significant soil carbon loss, eventually resulting in similar SR and organic carbon at all the elevations (Table 1). Neutrality in soil organic carbon towards lower elevations in our study (except at 3300 m) indicates a net-zero CO_2_ flux with rising temperature compared to previously stated increasing and decreasing carbon trends from other regions suggesting net-gain and net-loss in CO_2_ flux, respectively^7,13^. Though reported for the first time in our study, the equilibrium in SR across elevation gradient is evident in other studies also. For instance, Zhao et al. displayed comparable SR rates across most of their sites in an alpine meadow (4400–5200 m)^7^. Similarly, Ma et al. showed relatively stable SR and its components (autotrophic and heterotrophic respiration) across 3 out of 4 habitats in a sub-tropical forest, namely evergreen broad-leaved (780 m), mixed evergreen (1670 m), and deciduous broad-leaved (1970 m) forests^13^. We hypothesize that certain ecosystems may limit the warming-induced increase in SR by altering their edaphic factors, especially moisture, limiting soil carbon loss under sustained plant carbon inputs.

## Conclusion

The temperature-sensitive elevation gradient in our study demonstrate an equilibrium mechanism in CO_2_ emissions, making it resilient to rising temperatures and preventing soil carbon loss. Across the four-month sampling period, we find that SR response depends on the strength of the temperature-SR relationship, which depends on moisture availability in the soil. Higher temperatures and low soil moisture towards lower elevations reduced temperature sensitivity of SR causing a neutral response of SR to warming. Positive SR responses to increasing temperature, if observed, are primarily short-lived and limited by either moisture or substrate deficiency^36,37^. The equilibrium mechanism, suggesting net zero SR rates under climate change in this region, is vital in enhancing the climate change impact projections as it accounts for both SR and its governing factors simultaneously at different spatial and temporal scales. Moreover, climosequence can be used as a surrogate to capture the climate change triggered responses of SR and its sensitivity to rising temperature. A limitation of our study is the short SR measurement period which may have effects on its temperature sensitivity range.

## Methods

### Study site characteristics

The study was conducted across a 700 m elevation gradient (3300–4000 m) along the south-west facing slope of a glaciated valley in western Himalaya in Gangotri National Park, India (30° 56'–30° 59' N, 78° 58'–79° 3' E) (Supplementary Fig. S1 online). Vegetation along this elevation gradient can be broadly classified as subalpine forest (3300–3500 ± 200 m), alpine scrub (3500–3800 ± 200 m), and alpine meadow (3800 m and above) (Supplementary Table S1 online). Mean annual air temperature, recorded by data loggers during 2017–18, were 5.9 ± 0.4 °C, 3.7 ± 0.2 °C, and 1.6 ± 0.1 °C at 3300–3400 m, 3600–3700 m and 3800–3900 m elevation ranges, respectively. The elevation ranges hence provide a ~ 2.1 °C gradient in temperature, forming a climosequence. Mean annual precipitation is 1500 mm, occurring as rainfall from July to September and snowfall from December to May^38^. The soil in this region is relatively dry and poorly formed, with a thin upper organic layer mixed with gravel. Six elevations were selected across a 700 m elevation gradient (3300–4000 m), and six plots (0.5 × 0.5 m) were marked at each elevation at a distance of 3–5 m away from the trekking route. Study sites were relatively uniform in terms of aspect, slope, and soil taxonomy.

### Evaluating soil respiration at different elevations

At each plot, hollow cylindrical PVC collars (diameter: 20 cm and height = 12 cm) were inserted 2–3 cm inside soil. Plants inside the collars were clipped from above 1 mm ground without disturbing the soil. SR was measured from the collars using LI-8100A Automated Soil CO_2_ Flux System (LICOR, Inc., Lincoln, NE, USA) with an opaque survey chamber. Measurements were taken during the 2019 growing season twice each month from June to October (except August due to excess rainfall) on clear days during 0800–1600 h. The measurement dates were 19th and 23rd June, 13th and 17th July, 4th and 8th September, and 23rd and 27th October. The observation time was kept at 120 s with a dead band of 15 s.

### Soil temperature, moisture, and organic carbon measurements

Soil temperature and volumetric soil water content (SWC) at 5 cm depth were measured using a hand-held soil temperature probe (6000-09TC, LICOR Inc., Lincoln, NE, USA) and a GS1 soil moisture sensor (Decagon Devices, Inc., Pullman, WA) attached to the LI-8100A instrument. Soil samples were collected at 5 cm depth from each collar post-SR measurement using a soil auger (diameter 5 cm) and pooled in plastic zip-lock bags. The soil was homogenized, air-dried, sieved through a 1 mm sieve, and stored under 4 °C. Soil organic carbon was estimated in duplicates from 0.1 g soil using the potassium dichromate oxidation method^39^.

### Statistical analyses

The data's normal distribution and homogeneity of variance were determined through Shapiro–Wilk and Levene's test, respectively. Since the data did not meet the assumption of normality and homogeneity even after log transformation, we conducted Welch's analysis of variance (ANOVA) to assess differences in parameters among different elevations^40^. Where differences existed, we used the post-hoc Games-Howell test for pairwise comparisons. All means and differences were reported in terms of monthly mean ± standard error. The relationship between SR and soil temperature at different elevations was examined by fitting an exponential regression model

1 $${\text{SR}}\, = \,\alpha {\text{e}}^{{\beta {\text{t}}}}$$

where t is the soil temperature at 5 cm depth, the coefficient α is the intercept of respiration at 0 °C, and β represents the temperature sensitivity of SR.

Q_10_ of SR was calculated based on coefficient β as.

2 $${\text{Q}}_{{{1}0}} \, = \,{\text{e}}^{{{1}0}} \beta$$

Simple linear regression was conducted to determine the probable effect of SWC on SR at each elevation. In addition, linear relationships between Q_10_ of SR and soil temperature and moisture were also assessed. All statistical analyses were performed in SPSS 23.0 (IBM, Chicago, IL, USA), and significant differences were assessed at the level p < 0.05.

## Supplementary Information

Supplementary Information 1.

Supplementary Information 2.
