# Supplementary material for: Equilibrium in soil respiration across a climosequence indicates its resilience to climate change in a glaciated valley, western Himalaya
Source: Sci Rep. 2021 Nov 29;11:23038. doi: 10.1038/s41598-021-02199-x (PMC8630114; doi:10.1038/s41598-021-02199-x)
Supplement: Supplementary file 2 — Supplementary Information 2. [file 41598_2021_2199_MOESM2_ESM.docx]

**Equilibrium in soil respiration across a climosequence indicates its resilience to climate change in a glaciated valley, western Himalaya**

**Authors**: Pankaj Tiwari, Pamela Bhattacharya, Gopal Singh Rawat, Gautam Talukdar^*^

Wildlife Institute of India, Dehradun, Uttarakhand, India, 248001

**^*^Corresponding author**: [gautamtalukdar@gmail.com](mailto:gautamtalukdar@gmail.com), 0135-2646255


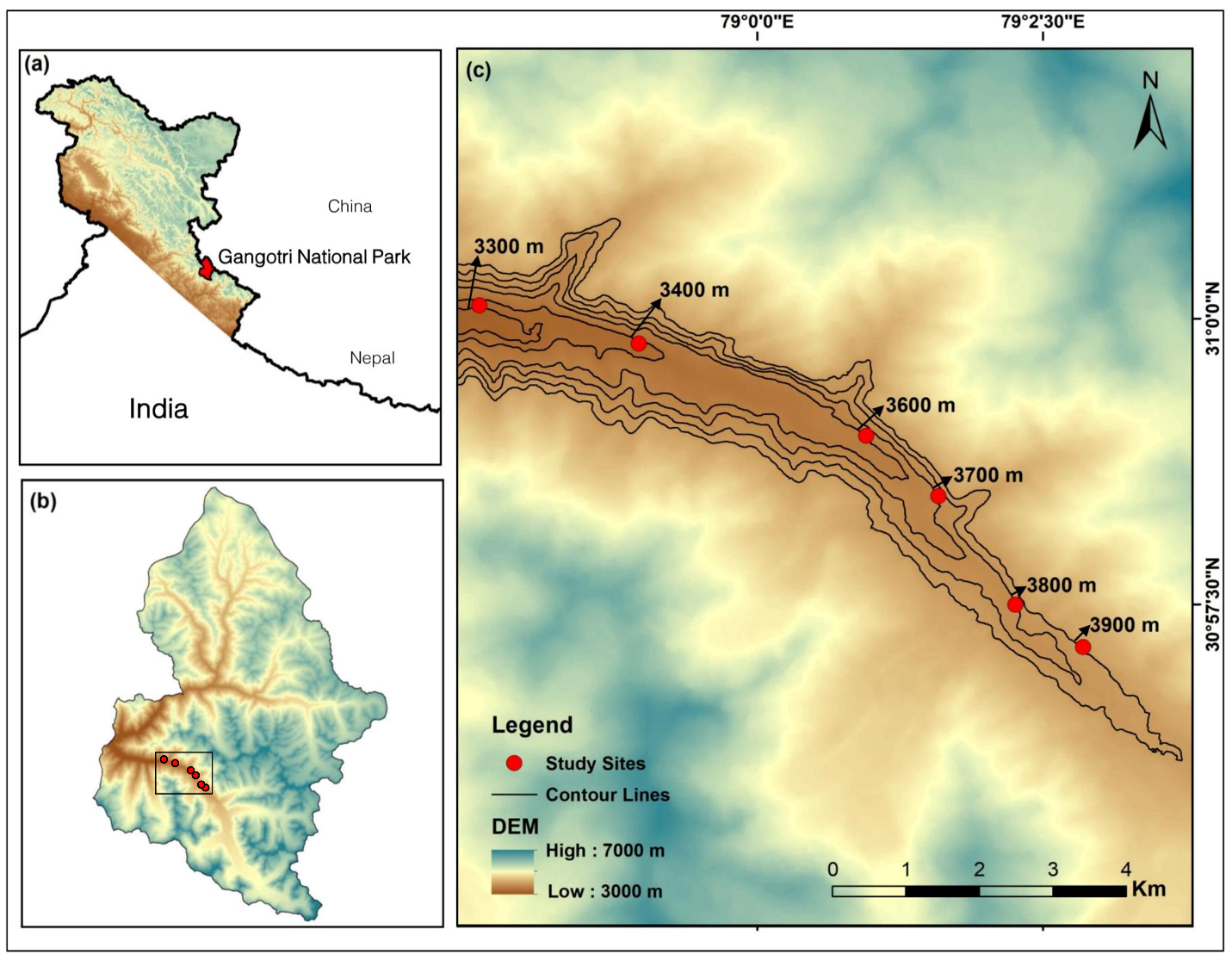


**Figure S1. Elevation sites in Himalaya** (a) Western Himalayan region in India with the boundary of Gangotri National park (b) Gangotri National Park with sampling sites and (c) Six study sites along an elevation gradient from 3300 m to 3900 m above sea level. Maps were generated in ArcGIS version 10.7 (ESRI, CA, USA, <https://desktop.arcgis.com/en/arcmap/>). 30 m SRTM DEM (Shuttle Radar Topography Mission Digital Elevation Model) data used in maps was downloaded from the US Geological Survey Earth Explorer (<https://earthexplorer.usgs.gov/>).

**Table S1. Broad profile of vegetation at different elevation range.** The table describes the type of vegetation with characteristic plant species at different elevation ranges at the study site. Note that there are no clear boundaries of vegetation at 3300-3600 and 3600-3800 m elevation ranges as depicted by ± symbol followed by the approximate spread distance.

| **Elevation (m a.s.l.)** | **Vegetation type** | **Habitat characteristics** | **Characteristic species** |
| --- | --- | --- | --- |
| 3300 – 3600 ± 200 | Sub-alpine Forest | South facing slope (25 – 35^o^) with scattered *Cedrus deodara* and *Pinus wallichiana*. Canopy cover ranging from 40 – 60% | Tree layer: *Cedrus deodara, Pinus wallichiana, Betula utilis, Populus ciliata, Prunus cornuta, Sorbus cuspidata*  Shrubs: *Ribes alpestre, Rosa sericea*, *Viburnum cotinifolium, Cotoneaster bacillaris, Lonicera quinquilocularis, Lonicera hypoleuca, Spiraea lindleyana*  Herbs*: Astragalus candolleanus, Rosularia alpestris, Anaphalis triplinervis, Chenopodium album, Lychnis fimbriata, Verascum Thapsus*  Graminoids: *Dactylis glomerata, Calamogrostis emodensis* |
| 3600 – 3800 ± 200 | Alpine Scrub | South-facing bouldery and rocky slopes (~35^o^)  Three broad communities are divisible (a) Riverine willow scrub, (b) Lower alpine scrub on the lower slopes, (c) *Artemisia* scrub on higher slopes | Riverine (Willow) scrub: *Salix dolichostachya, Myricaria elegans*  Lower alpine scrub: *Rosa sericea, Spiraea canescens, Lonicera myrtillus, Lonicera webbiana*  Artemisia scrub: *Artemisia santolinifolia, Artemisa dubia, Juniperus communis, Juniperus indica*  Ground vegetation (Herbaceous): *Astragalus candolleanus, Calamagrostis emodensis, Nepeta discolor, Festuca gigantea, Geranium himalayense, Hylotelephium ewersii, Aconogonum tortuosum, Senecio chrysanthemoides, Cynoglossum* sp.*, Bupleurum himalayense, Polygonatum cirrhifolium* |
| 3800 – 4000 | Alpine Meadow | South and south-west facing stable slopes (~35^o^). Broad classes include alpine herbaceous formations and *Kobresia* meadows | Herbaceous species*: Arenaria festucoides, Astragalus candoleanus, Rosularia alpestris, Anaphalis nepalensis, Nepeta discolor, Thymus serpyllum, Geranium himalayense, Sibbaldia cuneata, Erysimum hieraciifolium, Galium aparine, Origanum vulgare, Persicaria polystachya, Stipa* sp.  Graminoids: *Festuca valesiaca, Elymus nutans, Elymus sp., Stipa* spp.*, Kobresia nutans, Calamogrostis epigejos, Carex* spp. |

**Table S2. Regression models for SR, soil temperature and moisture.** Exponential (SR = αe^βt^) and linear models (SR = αM+β) to assess the relationships of SR to soil temperature (t) and volumetric soil water content (M) at each of the 6 altitudes during the growing season. α and β are constants and indicate intercept and slope of the relationship, respectively. Values in parentheses indicate standard error of the estimate.

| **Elevation (m a.s.l.)** | **a** | **b** | **r^2^** | **p** |
| --- | --- | --- | --- | --- |
| **SR= ae^bt^** |  |  |  |  |
| 3300 | 6.139 (3.864) | -0.076 (0.041) | 0.069 | 0.071 |
| 3400 | 0.907 (0.335) | 0.035 (0.025) | 0.041 | 0.167 |
| 3600 | 0.368 (0.178) | 0.099 (0.034) | 0.158 | 0.005 |
| 3700 | 0.421 (0.061) | 0.127 (0.012) | 0.721 | <0.001 |
| 3800 | 0.216 (0.048) | 0.160 (0.018) | 0.635 | <0.001 |
| 3900 | 0.324 (0.036) | 0.146 (0.009) | 0.862 | <0.001 |
|  |  |  |  |  |
| **SR= aM+b** | |  |  |  |
| 3300 | 0.966 (0.408) | 18.547 (4.789) | 0.246 | <0.001 |
| 3400 | 1.242 (0.296) | 11.560 (3.273) | 0.213 | <0.001 |
| 3600 | 1.304 (0.225) | 8.834 (2.133) | 0.272 | <0.001 |
| 3700 | 1.377 (0.325) | 7.012 (2.335) | 0.164 | 0.004 |
| 3800 | 0.985 (0.257) | 8.433 (1.732) | 0.340 | <0.001 |
| 3900 | 1.165 (0.311) | 11.858 (2.356) | 0.355 | <0.001 |
